# Investigation of the Possible Antibacterial Effects of Corticioid Fungi Against Different Bacterial Species

**DOI:** 10.3390/ijms26073292

**Published:** 2025-04-02

**Authors:** Eugene Yurchenko, Małgorzata Krasowska, Małgorzata Kowczyk-Sadowy, Ewa Zapora

**Affiliations:** 1 Institute of Forest Sciences, Białystok University of Technology, Wiejska St. 45E, 15-351 Białystok, Poland; e.zapora@pb.edu.pl; 2 Department of Agri-Food Engineering and Environmental Management, Białystok University of Technology, Wiejska St. 45E, 15-351 Białystok, Poland; m.krasowska@pb.edu.pl (M.K.); m.kowczyk@pb.edu.pl (M.K.-S.); 3 NatureTECH Centre of Natural Products Research, Białystok University of Technology, Wiejska St. 45E, 15-351 Białystok, Poland

**Keywords:** Agaricomycetes, antibacterial activity, Basidiomycota, corticioid fungi

## Abstract

Extracts from 58 species of corticioid fungi (phylum Basidiomycota), mainly belonging to the orders Hymenochaetales, Polyporales and Russulales, were tested for their inhibitory activity against five species of bacteria: *Corynebacterium striatum*, *Haemophilus influenzae*, *Klebsiella pneumoniae*, *Pseudomonas aeruginosa*, and *Staphylococcus aureus*. Twenty-four of the species we analyzed in this study were tested for antibacterial activity for the first time. The fruiting bodies of the fungi were collected from dead wood in the forests of north-eastern Poland, and macerated in methanol. Dried extracts were redissolved in dimethyl sulfoxide and applied to broth cultures of the bacteria, which were then inoculated on agar plates. *Noblesia crocea* demonstrated moderate inhibitory activity against all five tested bacteria; *Amylocorticium subincarnatum*, *Laxitextum bicolor*, *Peniophora laeta*, *P. rufomarginata*, *Phanerochaete sordida*, and *Xylobolus frustulatus* inhibited four bacterial species. The extracts from 14 fungal species tested were moderately active against only two bacteria, *P. aeruginosa* and *C. striatum*; 17 species were active against *C. striatum* only. The full inhibition was observed with concentrations of extract 25 or 50 mg/mL.

## 1. Introduction

Bacterial pathogens pose a significant threat to human health. The increasing prevalence of antibiotic-resistant strains underscores the urgent need for novel therapeutic strategies [1,2,3,4]. Research is increasingly focused on exploring solutions of natural origin to combat these formidable pathogens [4]. Some of the most serious bacteria include *Staphylococcus aureus*, *Klebsiella pneumoniae*, *Pseudomonas aeruginosa*, *Haemophilus influenzae*, and *Corynebacterium striatum*. They are notable for their association with serious respiratory infections, which are challenging to manage due to their ability to form biofilms and develop resistance to antibiotics [5,6,7,8].

The bacteria listed above can cause not only respiratory diseases, but also other health issues. *Staphylococcus aureus* is a Gram-positive opportunistic prokaryote that is a significant component of the respiratory microbiota [9]. It is a major agent of pneumonia, but can also cause sinusitis [10], skin and cardiovascular infections, sepsis, and nosocomial bacteremia [11]. *Klebsiella pneumoniae* is a Gram-negative bacterium that occurs naturally in the human digestive tract. In recent years, it has emerged as an important pathogen due to the increasing occurrence of hypervirulent and antibiotic-resistant strains. It causes infections of the lungs, urinary tract, bloodstream, wound or surgical site, and brain [12]. *Pseudomonas aeruginosa* is a Gram-negative opportunistic prokaryote found in humans mainly in the gastrointestinal tract or on the skin. It is a major component of the respiratory microbiota [9] and causes pneumonia, bacteremia, urinary tract and surgical infections [13]. *Haemophilus influenzae* is a Gram-negative bacterium that is transmitted by airborne droplets. It can cause sinusitis, pharyngitis [10], meningitis, otitis media, and septicemia [14]. *Corynebacterium striatum* is a Gram-positive, multi-drug resistant prokaryote that can cause nosocomial outbreaks. It is a part of the normal skin microbiota but can provoke bacteremia, endocarditis, and pneumonia under the right conditions [15,16].

The epidemiological challenges of nowadays, along with the fairly fast development of the resistance of bacteria to commercial antibiotics [17,18,19,20], is the cause for searching for new antibacterial substances. Fungi are considered to be a source of many as yet undiscovered antibacterial compounds for potential future use [21,22]. There is a prospect that some of the new antibacterial substances will not only be useful in treating bacterial infections. This is based on the fact that some substances known as antibacterial drugs have antiviral activity against SARS-CoV-2 and an immunomodulatory effect [23,24]. Deadwood-associated fungi have shown remarkable potential in the discovery of bioactive compounds with medicinal properties. These fungi are known to produce secondary metabolites with antimicrobial, antiviral, and anticancer activities, making them a valuable resource for drug development. Research into these fungi could provide solutions to pressing challenges such as antibiotic resistance.

Among Basidiomycota, corticioid fungi are an important group of wood-decaying organisms, found in all wood-containing ecosystems on Earth. They are particularly common on fallen wood, both small-sized and coarse, in temperate and boreal forests, and eventually participate in soil formation. Morphologically corticioid fungi are characterized by flat fruiting bodies (basidiomata), usually 0.05–0.5 mm thick and effused over the substratum, and bearing one-celled basidia [25]. However, in terms of natural classification, they belong to different orders of Agaricomycetes, with a few exceptions of the genera assigned to the classes Dacrymycetes and Tremellomycetes [26,27]. Wood-inhabiting Basidiomycota, including the corticioid fungi, compete with bacteria for low molecular weight nutrients in dead wood [28]. It has been demonstrated experimentally that the wood colonized by some corticioid fungi contains significantly fewer bacterial cells, than the wood without these fungi, as in the case of *Phlebiopsis gigantea* [29] and *Resinicium bicolor* [30]. Consequently, it is recognized that corticioid fungi can produce antibacterial compounds. Some of these substances have been isolated from the genera *Dentipellis*, *Merulius, Peniophora, Stereum*, and *Xylobolus* [31,32,33,34,35,36,37]. However, none of these compounds has yet been commercialized for medical application, and their mechanisms of action on the bacterial cell remain unknown.

There have been a number of pioneering papers on the antibacterial properties of extracts from the fruiting bodies of wild basidiomycetes, including three that were the first to study corticioid fungi. Wilking and Harris [38] screened 37 corticioid fungi, of which 9 species only demonstrated antibacterial properties. In the study by Mathieson [39], 13 species of corticoid fungi were tested and 3 of them showed antibacterial activity. Wilkins [40] studied 8 corticioid species, 2 of which were active against bacteria. In these studies, the authors obtained extracts from ground basidiocarps, with or without the addition of water, and applied them to the agar plates inoculated with bacteria. This type of experiment was considered to be a preliminary test, whereas the tests with fungi isolated in pure culture were more substantial.

In subsequent work, only basidiomata of sufficient thickness, length, and ease of removal from the substratum were used for antibacterial studies. Gianetti et al. [41] isolated the antibacterial substances: merulinic acids A, B, and C from the fruiting bodies of *Merulius tremellosus* and *Phlebia radiata*. Zjawiony et al. [42] proved that the ethanolic extract of the fruiting bodies of *Byssomerulius incarnatus* has antibacterial properties against *S. aureus*. Cateni et al. [43] extracted by methanol and purified four compounds from fresh *Stereum hirsutum* basidiomata, and reported the activity of compounds from this fungus against *Mycobacterium tuberculosis*. Ferreira-Silva et al. [44] found that ethyl acetate extract from *Stereum ostrea* basidiomata was active against *S. aureus*. Tamrakar et al. [45] found the antibacterial activity of ethanolic extracts from fruiting bodies of *Phlebia tremellosa* and *Xylobolus princeps*. Sevindik et al. [46] showed that ethanolic and methanolic extracts from fruiting bodies of *Stereum hirsutum* were active against five species of bacteria. İnci et al. [47] found that ethanolic extract from *Hymenochaete rubiginosa* basidiomata was active against five bacterial strains. For most species of corticioid fungi, which have thin basidiomata closely adnate to the wooden substratum, no antibacterial tests have been attempted since 1946.

In addition, a number of papers have been published after studying the antibacterial activities of pure cultures of corticioid fungi. In these cases, the live fungi or their derivatives were applied against bacteria in vitro. The mycelium of corticioid fungi growing on agar or agar fragments taken from the surrounding of the growing fungi have been used in screening studies [48,49,50,51,52,53,54]. The broth from the submerged culture of the fungi was used in the works [49,50,55]. Methanolic extracts of the whole submerged culture of the fungi were tested by Suay et al. [56]. Extracts from mycelia obtained after submerged cultivation were used by Grey et al. [54] and Rosa et al. [57]. Methanolic extracts from mycelium after solid-state cultivation were tested by Zrimec et al. [58].

The aim of this study was to screen the crude extracts of corticioid fungi, including 24 species for the first time, for antibacterial properties against the main bacteria known as agents of respiratory co-infections.

## 2. Results

The crude extracts involved in the research were yellow-brown or dark brown in color, with specific variations ranging from brownish-yellow in *Hyphoderma setigerum* to reddish-black in *Noblesia crocea* and almost pure black in *Boreostereum radiatum*. The consistency of the extracts was that of a soft resin or soft paste. The extraction yield depended on the ratio of the dry mass of the fungus to the volume of methanol, with significant differences between small and large weight samples. For samples from 0.6 to 3.0 g the yield was 8.8–60.4% with an average of 24.3%. For fungal samples from 3.1 to 75.0 g the yield ranged from 2.2 to 45.0% with an average of 11.4%. In the latter group, the highest yield was for *Byssomerulius corium* (27.9%), *Dentipellis fragilis* (28.2%), and *Noblesia crocea* (45.0%). Due to the variation in dry mass of the samples, size, and density of the basidiomata pieces, it was not realistic to use identical sample/solvent ratios (*w*/*w* or *v*/*v*) for all samples. In this respect, it was observed that the efficiency of extraction increased significantly with the decreasing material volume and increasing solvent volume up to 1:10 and higher.

Of the 58 fungal species tested, extracts from 51 species (88%) demonstrated total inhibition (at very high concentrations) of at least one bacterial strain (Table 1). No inhibition of bacterial growth was observed in solvent controls (DMSO).

The activity of 24 species of corticioid fungi against bacteria was tested for the first time. We also screened 58 fungal species for the first time for activity against *H. influenzae* and *C. striatum*.

*Amylocorticium subincarnatum*, *Laxitextum bicolor*, *Peniophora laeta*, *P. rufomarginata*, *Phanerochaete sordida*, and *Xylobolus frustulatus* moderately inhibited four bacterial strains. Fourteen corticioid species tested were only active against two bacteria: *P. aeruginosa* and *C. striatum*; 17 fungal species were only active against *C. striatum*; 7 species had no activity against bacteria in this assay.

Notable differences in activity between samples were observed in 12 species (Table 1). In *Phlebiopsis gigantea*, the winter collected sample with older basidiomata (No. 247-1) was more active, than the spring one with younger basidiomata (No. 247-2). In *Byssomerulius corium*, the spring collected sample with older basidiomata (No. 264) was active, whereas the autumn sample with younger basidiomata (No. 245) was inactive. In *Xylobolus frustulatus*, the summer sample (No. 186-1) was more active than the spring one (No. 186-2). In *Resinicium bicolor,* the autumn collection (No. 308) was more active than spring one (No. 276). In *Peniophora rufomarginata*, the autumn sample (No. 249-2) was more active, than the winter one (No. 249-1). In *Baltazaria galactina*, the autumn sample (No. 233) was active, whereas the summer one (No. 231) was inactive.

For some samples, the influence of the substratum was also admitted. For example, *Baltazaria galactina* was active in the case of the sample from *Tilia cordata* (No. 233) and inactive in the sample from *Populus tremula* (No. 231). *Byssomerulius corium* collected from *Carpinus betulus* (No. 264) showed moderate activity, but that collected from *Populus tremula* (No. 245) was inactive. *Resinicium bicolor* collected from *Pinus sylvestris* (No. 308) was active, but that one collected from *Picea abies* (No. 276) was inactive. For *Lyomyces crustosus* only, no variation in activity between samples was detected.

The distribution of activities among the members of one genus was, according to our results, rather uneven. For example, the activity was not detected in *Amylocorticium cebennense*, whereas it was remarkable in *A. subincarnatum*. A similar contrast is observed between *Phanerochaete velutina* and *Ph. sordida*, *Peniophora quercina* and *P. rufomarginata*. For the genera *Hymenochaete*, *Hyphoderma*, *Stereum*, and *Xylodon* the patterns of moderate activity are similar for species within a genus.

**Figure 1:**
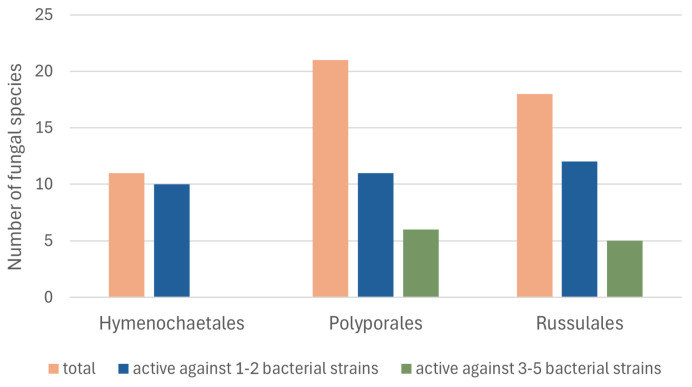
Distribution of antibacterial activity according to fungal order.

The sample collection was focused on three orders of the fungi, which allows for tracing a distribution pattern of antibacterial activities at the order level (Figure 1). Taking into account the number of sensitive bacterial strains, moderate screening results belonged to the Polyporales and the Russulales. The activities of the Hymenochaetales species were limited to less sensitive bacterial strains.

**Figure 2:**
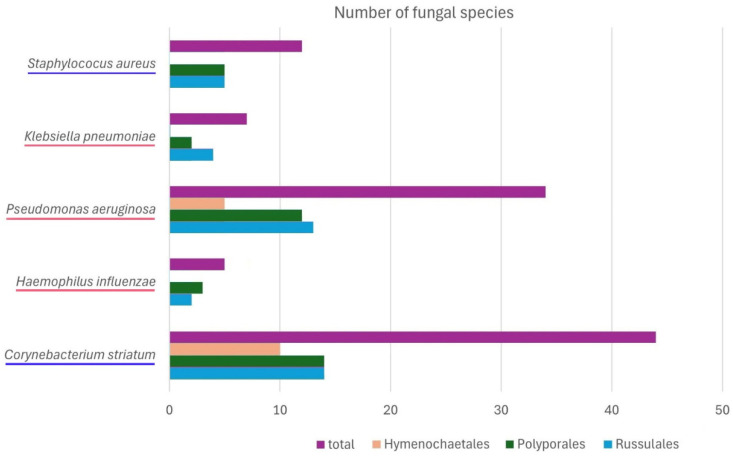
Susceptibility of the bacterial strains—the number of fungal species for which inhibition of the strains by at least one of the samples tested was observed. Names of the Gram-positive strains are underlined in blue and the Gram-negative strains in pink.

A clear pattern of differential susceptibility of bacterial strains to the fungal metabolites can be seen in the present study. This can be described through the number of fungal species that moderately inhibit the growth of each strain of bacteria (Figure 2). Namely, *C. striatum* and *P. aeruginosa* were found to be more susceptible, whereas *S. aureus*, *K. pneumoniae*, and *H. influenzae* were more resistant. No dependence of susceptibility on the type of fungal cell wall (Gram-positive or Gram-negative) was observed in our study.

## 3. Discussion

This study demonstrated that fungal metabolites have a moderate effect on different bacteria than would be expected based on the microbiology of their habitats. This is evident from the fact that human pathogenic bacteria from the list of strains tested above are not known from dead wood in forests. However, studies by molecular methods have shown that the genus *Pseudomonas* is common in dead wood [29,59,60]. In addition, a *Staphylococcus species* related to *S. aureus* was recorded in dead wood [30]. Thus, fungi possess bactericidal mechanisms not only against their direct competitors but also against relatives of such competitive bacteria.

Although the activity of the tested fungi was not so sufficient to make them promising as antibiotic agents, our study showed that the percentage of more active species was higher in the orders Polyporales and Russulales. We assume that in the Russulales, such activities are associated with the presence of gloeocystidia as a receptacle of secondary metabolites, like in *Laxitextum*, *Peniophora*, and *Xylobolus*, whereas in the Polyporales, the association of active substances with the anatomical structures of the basidioma has not yet been hypothesized.

A remarkable phenomenon is that the activity against a bacterial strain is not repeated for different samples belonging to the same fungal species. This inconsistency has already been noted in a pioneer work [38] for fruiting bodies collected in different years and different locations. The activity was also determined to be strain-dependent in tests with fungi isolated in pure culture [48,50,56].

Apart from the ontogenesis of the vegetative body, individual fruiting bodies of corticioid fungi have their developmental stages from primordia to the collapse of hymenial elements and total destruction of the basidioma. Using younger and older basidiomata for extraction we consider as a cause of different antibacterial activities manifestation between samples of the same species. Different ontogenetic stages of the samples were also a supposed reason for the different colors of crude extracts for the same species. Furthermore, we assume that different proportions of sample/solvent weight during maceration can be a cause of differences in active substance concentration between samples. No apparent pattern of activity dependence on the season of fruitbody collection was observed, but a larger number of samples of individual species from the wild are required for reasoned conclusions.

Eighteen species from our study were previously tested for activity against *S. aureus* in three pioneering papers, based on basidiomata collected in situ [38,39,40]. In these experiments, extracts were prepared as fungal homogenate in distilled water 1:1 *w*/*w* [39], or the liquid, naturally present in the fresh fruiting body, was squeezed out [38,40]. However, for the second case, the articles do not give details of how the liquid was obtained for basidiomata thinner than 0.5 mm. Overall, only two fungal species from our study showed moderate activity against *S. aureus*, as in these publications: *Byssomerulius corium* [39] and *Phlebiopsis gigantea* [38]. The activity against *S. aureus* reported for *Hyphoderma setigerum*, *Stereum sanguinolentum* [38], *Phlebia rufa*, and *Scopuloides hydnoides* [40] was not confirmed in our research. Contrary to our results, the above authors did not report activity against *S. aureus* in *Chondrostereum purpureum* and *Peniophora cinerea*.

Screening for antimicrobial properties of fungi using the material from in situ-developed fruiting bodies can be a first step before trials with their pure cultures. At the same time, we acknowledge that wild-growing basidiomata may contain a complex of substances with antibacterial activity that is not produced in the same quality and quantity as mycelium grown in vitro.

We, therefore, compared our results with those of tests against *S. aureus* using pure living cultures of corticioid fungi [48,50]. Three summaries can be distinguished as follows:

(1) The fungal species that inhibited the growth of *S. aureus* both in our experiments and as mycelia growing in culture are the following: *Baltazaria galactina*, *Byssomerulius corium*, *Chondrostereum purpureum*, *Laxitextum bicolor*, *Noblesia crocea*, *Peniophora cinerea*, and *Xylobolus frustulatus*;
(2) The fungal species that inhibited the growth of *S. aureus* as fruiting body extracts, but did not show such activity as mycelia in culture are the following: *Phanerochaete sordida* and *Phlebiopsis gigantea*.
(3) The fungal species that were not active in their fruiting body extracts but were active as living mycelia are the following: *Hydnoporia tabacina*, *Hymenochaete rubiginosa*, *Mycoacia livida*, *Peniophora incarnata*, *P. quercina*, *Stereum hirsutum*, *S. rugosum*, and *S. sanguinolentum*.

The same comparison was conducted with published data on the inhibitory activity of extracts from cultures against *S. aureus* [55,56]. In this case, two groups of species can be distinguished as follows:

(1) The fungi that inhibited *S. aureus* as fruitbody extracts, but not as culture extracts are the following: *Chondrostereum purpureum*, *Irpex lacteus*, and *Xylobolus frustulatus*;
(2) The fungi that were not active as fruitbody extracts, but were active as culture extracts are the following: *Peniophora quercina* and *Stereum hirsutum*.

In some sources, the activity of corticioid fungi against *P. aeruginosa*, based on basidiomata extracts, has been studied; these data are similar to our results. Namely, it was found that ethanolic extract of *Hymenochaete rubiginosa* had low antibacterial effect against this bacterium with minimum inhibitory concentration (MIC) = 200 mg/mL [47]; ethanolic and methanolic extracts of *Stereum hirsutum* inhibited this bacterium with MIC = 100 mg/mL [46].

There are experimental data on extracts from cultured mycelia tested against *P. aeruginosa* [56,57], and the species from these experiments can be divided into the following four groups:

(1) Active both in our study and in the study [56]: *Chondrostereum purpureum*;
(2) Active in our study, not or very little active in the studies [56,57]: *Byssomerulius corium*, *Irpex lacteus*, *Peniophora cinerea*, *P. incarnata*, *P. limitata*, and *Steccherinum ochraceum*;
(3) Highly active in the study [56], not active in our study: *Coniophora arida* and *Peniophora quercina*;
(4) Not active in our study and in the study [56]: *Stereum hirsutum* and *Xylodon paradoxus*.

The activity of corticioid fungi against *K. pneumoniae* has been studied very poorly earlier. There are data that filtrate from the culture of *Stereum hirsutum* moderately inhibited this bacterium [61].

Our research showed that the percentage of species whose fruiting bodies contain substances that inhibit, although at very high concentrations, the growth of clinically important pathogenic bacteria is high among corticioid fungi. Based on the taxonomic analysis of the species list, the orders Polyporales and Russulales in general, and the genera *Amylocorticium*, *Peniophora*, *Phanerochaete*, and *Phlebia* in particular, are the perspective taxa for further searches of antibacterial substances. However, the antibacterial activity of fungal species based on methanol-soluble compounds from basidiomata collected in the wild, is not constant and is influenced by a number of factors that have not yet been clearly defined. There are differences in activity ranges and MBCs for individual bacteria between fungal samples collected in different seasons, from different hosts, and at different developmental stages of the basidiomata. In some cases, one sample showed activity, although at a very high concentration, against 2–3 bacterial strains, whereas another sample of the same species showed no activity.

## 4. Materials and Methods

### 4.1. Fungal Samples

The study included 84 samples belonging to 58 species of corticioid fungi. The fruiting bodies of the fungi were collected in fresh or partially dried (in dry weather) states from forests in the north-eastern part of Poland, mainly from the Białowieża Primeval Forest, in all seasons of the years 2017–2023. Most of the extracts were accompanied by the reference herbarium specimens of the fungi from which they were obtained. The reference specimens were deposited in Białystok University of Technology Herbarium–BLS (Appendix A, Table A1). Fungal samples collected by E. Yurchenko were identified by the same author. The names of the fungi are according to MycoBank (https://www.mycobank.org; accessed on 18 October 2024), and the order-level classification follows [27]. It was assumed that depending on the fungal species and the size of the substratum, the fruiting bodies of one sample came from one or more individual mycelia. In the latter case, the mycelia were from one or more substratum units, but the units were spatially close to each other. In the case of species producing abundant hyphal cords or rhizomorphs (*Etheirodon fimbriatum* and *Phanerochaete velutina*), these structures were taken for extraction together with hymenium-bearing parts.

### 4.2. Extract Preparation

After collecting in the field, the fruiting bodies were checked for colonization by other fungi. The fructifications infected by mycophilous fungi, mixed with other corticioid species, or those in a post-mature (destroyed) state were not used for extraction. In the laboratory, the fruiting bodies were separated with a scalpel, avoiding as far as possible the collection of substratum material, i.e., dead wood or bark. For this procedure, fruiting bodies were rehydrated in a moist chamber, if necessary. Thin fruiting bodies were usually detached from the substratum in fragments of 2–5 mm in size. Large fruiting bodies were cut into 1–3 cm pieces. The dry mass of the fungal material prepared in this way ranged from 0.6 g (*Amylocorticium cebennense*) to 75 g (*Noblesia crocea*) per sample.

The collected fungal material was dried at room temperature, weighed, and immersed in methanol (99.8%; Poch Basic—Avantor Performance Materials, Gliwice, Poland) in a ratio of 1:3 (*v*/*v*) for larger fungal samples and up to 1:10 (*v*/*v*) for smaller samples, in such a way that the fungal material was completely covered by the solvent. Methanol was chosen as the most effective solvent for obtaining extracts with antibacterial potential [62]. Passive extraction without stirring or shaking took 2 months in the dark at room temperature. In case when the amount of crude extract was too small, a second maceration of the same sample in methanol was carried out for 2 weeks. If the second tincture was dark pigmented, it was evaporated and added to the primary extract.

The finished tinctures were filtered through the 80 g/m^2^ filter paper and the solvent was evaporated in two steps. In the first step, most of the solvent was removed in a rotary evaporator Rotavapor^®^ R-100 (Büchi, Flawil, Switzerland) at 46 °C, rotation speed 3–4 units, and under the reduced pressure from 300 to 100 mbar. In the second step, the extract was collected from the walls of the extraction round-bottomed flask together with a small amount of methanol and placed in the glass extraction cells of a system of parallel evaporation Multivapor^TM^ P-12 (Büchi), where incubated at 46 °C and under the reduced pressure from 400 to 100 mbar. Each solid-state extract was stored in the dark at 10 °C in the Fungi Extract Bank^®^ collection (https://fungiextractbank.com/en, accessed on 17 February 2025; Appendix A, Table A1).

### 4.3. Bacterial Strains and Testing the Antibacterial Activity

Strains of bacteria *Corynebacterium striatum* PCM 3067, *Klebsiella pneumoniae* PCM 2713, *Pseudomonas aeruginosa* PCM 2270, and *Staphylococcus aureus* PCM 2267 were obtained from the Polish Collection of Microorganisms (Hirszfeld Institute of Immunology and Experimental Therapy, Wrocław, Poland). The strain *Haemophilus influenzae* (b) ATCC^®^ 10211 originated from the American Type Culture Collection (Rockville, MD, USA). The bacterial cultures in Mueller-Hinton broth (Merck, Darmstadt, Germany), 24 h post inoculation, with the density of suspension about 1 × 10^7^ colony-forming units/mL, were used as subsequent inocula in the experiments. The bacterial density was determined using the plate method, and inocula were diluted by physiological solution if needed. Liquid inoculum and test cultures of *H. influenzae* were maintained in tryptic soy broth with hemin and NAD, in microaerophilic conditions (6% CO_2_). All incubations of bacteria in liquid culture or on agar plates were carried out for 24 h at 37 ± 1 °C.

The antibacterial activity of the fungal extracts was preliminary assessed in glass tubes. Prior to this, the dried extracts were dissolved in dimethyl sulfoxide (DMSO; Merck) at a concentration of 100 mg/mL. DMSO was selected as a good amphiphilic solvent [63], which is not very toxic to bacteria in dilute concentrations [64,65]. These dissolved extracts were then diluted with distilled water to obtain the two test concentrations, 25 mg/mL and 50 mg/mL [66], and stored at 4 °C in dark glass bottles before the inoculation with bacteria. Each test tube contained 1.5 mL of broth, 0.5 mL of the tested extract 100 mg/mL, and 0.1 mL of broth inoculum (for the test concentration 25 mg/mL) or 1 mL of broth, 1 mL of the tested extract, and 0.1 mL of broth inoculum (for the test concentration 50 mg/mL). Solvent controls consisted of 1 mL of broth, 1 mL DMSO, and 0.1 mL of inoculum. Reference samples were the broth inoculated with bacteria. Tubes were visually inspected and those with no obvious bacterial growth, i.e., where the medium was not turbid, were considered to have the minimum inhibitory concentration (MIC) of the extract if this concentration followed the lower concentration at which growth was visible.

To check for growth inhibition, the second set of experiments was performed as a microdilution assay in wells of plastic 96-well microplates (Corning^®^, Corning, NY, USA), but in this case, the combinations of each strain with each extract in two concentrations (25 and 50 mg/mL) were repeated in four wells. Each well contained 100 μL of broth inoculum and 100 μL of dissolved extract. Negative controls contained 100 μL of broth inoculum and 100 μL of distilled water. Solvent controls contained 100 μL of broth inoculum and 100 μL DMSO. Controls were repeated in tetraplicate.

In the third set of experiments, the minimum bactericidal concentration (MBC) was determined from cultures on agar plates. The microplate wells in which no growth of a given microorganism was observed, i.e., with transparent contents, were selected, and a loopful of liquid was taken from them and streaked in a zig-zag pattern on agar plates in 90 mm Petri dish divided into 8 sectors. The media used were Mueller-Hinton agar (Merck) for *C. striatum*, *K. pneumoniae*, *P. aeruginosa*, *S. aureus*, and chocolate agar with polyvitamin supplement and bacitracin (Bio-Rad, Hercules, CA, USA) for *H. influenzae*. The MBC value was the concentration of the extract at which no bacterial growth was observed on agar.

## Figures and Tables

**Table 1:** Inhibitory activity of corticioid fungi extracts against selected bacteria.

O *	Fungal Species **	No. of Extract in the Fungi Extract Bank^®^	Inhibitory Effect of Fungal Extracts (Minimum Bactericidal Concentration, mg/mL)				
			*Staphylococcus aureus*	*Klebsiella* *pneumoniae*	*Pseudomonas* *aeruginosa*	*Haemophilus* *influenzae*	*Corynebacterium striatum*
Ag	*Chondrostereum purpureum* (Pers.) Pouzar	238	25	–	25	–	25
Ag	*Ch. purpureum*	248	–	–	25	–	–
Ag	***Radulomyces molaris*** (Chaillet ex Fr.) M.P. Christ.	239	–	–	25	–	–
Am	***Amylocorticium cebennense*** (Bourdot) Pouzar	344	–	–	–	–	–
Am	***A. subincarnatum*** (Peck) Pouzar	336	25	25	50	–	50
Am	***Irpicodon pendulus*** (Alb. & Schwein.) Pouzar	346	–	–	–	–	50
Ca	***Botryobasidium subcoronatum*** (Höhn. & Litsch.) Donk	303	–	–	–	–	25
Co	*Coniophora arida* (Fr.) P. Karst.	345	–	–	–	–	50
G	***Boreostereum radiatum*** (Peck) Parmasto	236	–	–	25	–	25
H	*Hydnoporia tabacina* (Sowerby) Spirin, Miettinen & K.H. Larss.	78	–	–	50	–	50
H	*H. tabacina*	243	–	–	–	–	–
H	*H. tabacina*	286	–	–	–	–	50
H	*Hymenochaete rubiginosa* (J.F. Gmel.) Lév.	56	–	–	50	–	50
H	***Kneiffiella barba-jovis*** (Bull.) P. Karst.	283	–	–	–	–	25
H	***Lyomyces crustosus*** (Pers.) P. Karst.	292-1	–	–	–	–	25
H	***L. crustosus***	292-2	–	–	–	–	25
H	*Peniophorella praetermissa* (P. Karst.) K.H. Larss.	349	–	–	–	–	–
H	*Resinicium bicolor* (Alb. & Schwein.) Parmasto	276	–	–	–	–	–
H	*R. bicolor*	308	–	–	50	–	50
H	***Skvortzovia furfuracea*** (Bres.) G. Gruhn & Hallenberg	285	–	–	–	–	50
H	***Xylodon brevisetus*** (P. Karst.) Hjortstam & Ryvarden	309	–	–	–	–	50
H	***Xylodon nesporii*** (Bres.) Hjortstam & Ryvarden	304	–	–	25	–	25
H	*Xylodon paradoxus* (Schrad.) Chevall.	265	–	–	–	–	50
H	*X. paradoxus*	284	–	–	–	–	25
H	*Xylodon spathulatus* (Schrad.) Kuntze	347	–	–	50	–	50
P	*Byssomerulius corium* (Pers.) Parmasto	245	–	–	–	–	–
P	*B. corium*	264	25	–	25	–	25
P	***Crustoderma dryinum*** (Berk. & M.A. Curtis) Parmasto	271	–	–	25	–	25
P	***Dacryobolus karstenii*** (Bres.) Oberw. ex Parmasto	348	–	–	–	–	50
P	***Etheirodon fimbriatum*** (Pers.) Banker	333	–	–	–	–	–
P	***E. fimbriatum***	341	–	–	–	–	–
P	***Hyphoderma transiens*** (Bres.) Parmasto	343	–	–	–	–	50
P	*Hyphoderma setigerum* (Fr.) Donk	222-1	–	–	–	–	–
P	*H. setigerum*	222-2	–	–	–	–	–
P	*Irpex lacteus* (Fr.) Fr.	311	25	–	50	–	50
P	***Meruliopsis taxicola*** (Pers.) Bondartsev	273	–	–	25	–	25
P	*Merulius tremellosus* Fr.	170	–	–	25	–	–
P	***Mutatoderma mutatum*** (Peck) C.E. Gómez	244	–	–	–	–	–
P	*Mycoacia livida* (Pers.) Zmitr.	335	–	–	–	–	50
P	*Noblesia crocea* (Schwein.) Nakasone	302-1	25	25	25	25	25
P	*N. crocea*	302-2	25	–	25	25	25
P	*Phanerochaete sordida* (P. Karst.) J. Erikss. & Ryvarden	291	25	25	25	–	25
P	*Ph. sordida*	305	–	–	25	–	25
P	*Phanerochaete velutina* (DC.) P. Karst.	334	–	–	–	–	25
P	*Phlebia centrifuga* P. Karst.	202-1	–	–	50	25	25
P	*Ph. centrifuga*	202-2	–	–	25	50	25
P	*Phlebia rufa* (Pers.) M.P. Christ.	229	–	–	50	50	–
P	*Ph. rufa*	232	–	–	–	–	–
P	***Phlebiodontia* cf. *subochracea*** (Bres.) Motato-Vásq. & Gugliotta	313	–	–	–	–	–
P	*Phlebiopsis gigantea* (Fr.) Jülich	247-1	25	–	25	–	25
P	*Ph. gigantea*	247-2	–	–	25	–	–
P	*Scopuloides hydnoides* (Cooke & Massee) Hjortstam & Ryvarden	338	–	–	50	–	–
P	***Steccherinum bourdotii*** Saliba & A. David	339	–	–	–	–	50
P	*Steccherinum ochraceum* (Pers. ex J.F. Gmel.) Gray	296	–	–	–	–	25
P	*S. ochraceum*	340	–	–	50	–	50
R	***Asterostroma medium*** Bres.	312	–	–	–	–	–
R	*Baltazaria galactina* (Fr.) Leal-Dutra, Dentinger & G.W. Griff.	231	–	–	–	–	–
R	*B. galactina*	233	25	–	50	–	–
R	*Dentipellis fragilis* (Pers.) Donk	342	–	–	50	–	–
R	***Gloiothele lactescens*** (Berk.) Hjortstam	314	–	–	25	–	–
R	*Laxitextum bicolor* (Pers.) Lentz	178	–	–	50	–	25
R	*L. bicolor*	235	25	25	25	–	25
R	*Peniophora cinerea* (Pers.) Cooke	293	–	–	–	–	50
R	*P. cinerea*	295	25	–	25	–	25
R	*P. cinerea*	310	–	–	–	–	50
R	*Peniophora incarnata* (Pers.) P. Karst.	272	–	–	50	–	25
R	*Peniophora laeta* (Fr.) Donk	288-1	–	25	25	–	25
R	*P. laeta*	288-2	–	25	25	25	25
R	*Peniophora limitata* (Chaillet ex Fr.) Cooke	290	–	–	25	–	25
R	***Peniophora pithya*** (Pers.) J. Erikss.	287	–	–	25	–	25
R	*Peniophora quercina* (Pers.) Cooke	237	–	–	–	–	50
R	***Peniophora rufomarginata*** (Pers.) Bourdot & Galzin	249-1	–	50	25	–	–
R	***P. rufomarginata***	249-2	25	–	25	25	25
R	***Scytinostroma odoratum*** (Fr.) Donk	337	–	–	25	–	25
R	*Stereum hirsutum* (Willd.) Pers.	289	–	–	–	–	25
R	*S. hirsutum*	294-1	–	–	–	–	–
R	*S. hirsutum*	294-2	–	–	–	–	–
R	*S. hirsutum*	167	–	–	–	–	–
R	*Stereum rugosum* Pers.	79	–	–	–	–	50
R	*S. rugosum*	281	–	–	–	–	–
R	*Stereum sanguinolentum* (Alb. & Schwein.) Fr.	274	–	–	25	–	25
R	*S. sanguinolentum*	275	–	–	–	–	50
R	*Stereum subtomentosum* Pouzar	197	–	–	–	–	25
R	*Xylobolus frustulatus* (Pers.) Boidin	186-1	25	50	25	–	25
R	*X. frustulatus*	186-2	25	–	–	–	25

* O = Order: Ag—Agaricales; Am—Amylocorticiales; Ca—Cantharellales; Co—Coniophorales; G—Gloeophyllales; H—Hymenochaetales; P—Polyporales; R—Russulales. ** names of the species tested for the first time for their antibacterial activity are shown in bold. The most active among the examined species was *Noblesia crocea*, which moderately inhibited the growth of all the bacteria tested. The basidiomata of this fungus were characterized by the ability to produce abundant reddish-black extract. Two extracts were obtained for this specimen: one from pieces of basidiomata free of substratum particles (extract No. 302-1), and the other from the basal parts of basidiomata interspersed with pieces of substratum (apple tree bark; No. 302-2). The preparation without bark showed a very high extract yield (45%), whereas the preparation with bark particles showed a yield of 24%. These extracts had similar activity patterns except for the absence of activity against *K. pneumoniae* in extract No. 302-2. As the basidiomata tissue of this species is yellow both when fresh and when dry, the black color of the extract appears to be a result of interaction with the solvent.

## Data Availability

Data is contained within the article.

## Appendix A

**Table A1:** Sample and extract data for the research.

Fungal Species	No. of Extract in the Fungi Extract Bank^®^	Collector(s) *, Month and Year of Collecting	Reference Herbarium Specimen No./Field Number	Area, Host
Agaricales				
*Chondrostereum purpureum*	238	KW, XI.2022		Hajnówka vicinities, *Pyrus domestica*
*Ch. purpureum*	248	EY, KW, XII.2022	BLS M-3975/EYu 221229-1	Hajnówka vicinities, *Cerasus vulgaris*
*Radulomyces molaris*	239	EY, V.2023	BLS M-10053/EYu 230509-6	BPF **, *Salix caprea*
Amylocorticiales				
*Amylocorticium cebennense*	344	EY, IX.2023	BLS M-10832/EYu 230926-8	BPF, *Picea abies*
*Amylocorticium subincarnatum*	336	EY, IX.2023	BLS M-10840/EYu 230926-1a	BPF, *Picea abies*
*Irpicodon pendulus*	346	EY, MW, XI.2023	BLS M-10831/EYu 231103-3	BPF, *Pinus sylvestris*
Cantharellales				
*Botryobasidium subcoronatum*	303	EY, V.2023	BLS M-10821/EYu 230525-3	BPF, *Pinus sylvestris*
Coniophorales				
*Coniophora arida*	345	EY, XI.2023	BLS M-10830/EYu 231103-2	BPF, *Picea abies*
Gloeophyllales				
*Boreostereum radiatum*	236	MW, X.2022	BLS M-3982	Białystok vicinities, *Picea abies*
Hymenochaetales				
*Hydnoporia tabacina*	78	EY, KW, XII.2022	BLS M-3974/EYu 221229-5	Hajnówka vicinities, *Corylus avellana*
*H. tabacina*	243	MW, X.2018	BLS M-603	BPF, *Salix cinerea*
*H. tabacina*	286	EY, VI.2023	BLS M-10046/EYu 230608-3	BPF, *Picea abies*
*Hymenochaete rubiginosa*	56	MW, XI.2021		BPF, *Quercus robur*
*Kneiffiella barba-jovis*	283	EY, VI.2023	BLS M-10064/EYu 230608-2	BPF, *Betula* sp.
*Lyomyces crustosus*	292-1	EY, VI.2023	BLS M-10311/EYu 230625-4	BPF, *Corylus avellana*
*L. crustosus*	292-2	EY, VIII.2023	BLS M-10323/EYu 230803-1	BPF, *Corylus avellana*
*Peniophorella praetermissa*	349	EY, IX.2023	BLS M-10842/EYu 230921-4	BPF, *Fraxinus excelsior*
*Resinicium bicolor*	276	EY, V.2023	BLS M-10054/EYu 230501-1	BPF, *Picea abies*
*R. bicolor*	308	EY, IX.2023	BLS M-11611/EYu 230907-2	BPF, *Pinus sylvestris*
*Skvortzovia furfuracea*	285	EY, V.2023	BLS M-10051/EYu 230509-4	BPF, *Pinus sylvestris*
*Xylodon brevisetus*	309	EY, IX.2023	BLS M-11612/EYu 230907-7	BPF, *Picea abies*
*Xylodon nesporii*	304	EY, VII.2023	BLS M-10321/EYu 230710-5	BPF, *Corylus avellana*
*Xylodon paradoxus*	265	EY, V.2023	BLS M-10050/EYu 230524-4	BPF, *Carpinus betulus*
*X. paradoxus*	284	EY, V.2023	BLS M-10048/EYu 230501-4	BPF, *Quercus robur*
*Xylodon spathulatus*	347	EY, MW, IX.2023	BLS M-10847/EYu 230919-6	Łuków vicinities, *Abies alba*
Polyporales				
*Byssomerulius corium*	245	EY, X.2022	BLS M-3939/EYu 221009-9	BPF, *Populus tremula*
*B. corium*	264	EY, IV.2023	BLS M-10063/EYu 230423-1	BPF, *Carpinus betulus*
*Crustoderma dryinum*	271	EY, V.2023	BLS M-10056/EYu 230511-1	BPF, *Picea abies*
*Dacryobolus karstenii*	348	EY, MW, XI.2023	BLS M-10829/EYu 231103-1	BPF, *Pinus sylvestris*
*Etheirodon fimbriatum*	333	EY, IX.2023	BLS M-10838/EYu 230926-2	BPF, *Alnus glutinosa*
*E. fimbriatum*	341	EY, X.2023	BLS M-10824/EYu 231008-4	BPF, *Salix caprea*
*Hyphoderma transiens*	343	EY X.2023	BLS M-10826/EYu 231008-6	BPF, *Tilia cordata*
*Hyphoderma setigerum*	222-1	EY, X.2022	BLS M-3944/EYu 221016-1	BPF, *Betula pendula*
*H. setigerum*	222-2	EY, III.2022	BLS M-3931/EYu 220320-1	BPF, *Betula pendula*
*Irpex lacteus*	311	EY, IX.2023	BLS M-10856/EYu 230907-6	BPF, *Sorbus aucuparia*
*Meruliopsis taxicola*	273	EY, MW, V.2023	BLS M-10060/EYu 230525-2	BPF, *Pinus sylvestris*
*Merulius tremellosus*	170	MW, XI.2019		BPF, *Populus tremula*
*Mutatoderma mutatum*	244	EY, X.2022	BLS M-3943/EYu 221009-8	BPF, *Populus tremula*
*Mycoacia livida*	335	EY, IX.2023	BLS M-10843/EYu 230921-3	BPF, *Fraxinus excelsior*
*Noblesia crocea* (without substratum)	302-1	MW, VIII.2023	BLS M-10309/230815-1a	Hajnówka vicinities, *Malus domestica*
*N. crocea* (the part of basidiomata interspersed with substratum particles)	302-2	MW, VIII.2023	BLS M-10309/230815-1b	the same as above
*Phanerochaete sordida*	291	EY, VI.2023	BLS M-10314/EYu 230625-5	BPF, *Fraxinus excelsior*
*Ph. sordida*	305	EY, VII.2023	BLS M-10322/EYu 230710-4	BPF, *Populus tremula*
*Phanerochaete velutina*	334	EY, IX.2023	BLS M-10844/EYu 230921-2	BPF, *Fraxinus excelsior*
*Phlebia centrifuga*	202-1	GK, VIII.2020		BPF, *Picea abies*
*Ph. centrifuga*	202-2	EY, X.2023	BLS M-3956/EYu 221016-7	BPF, *Picea abies*
*Phlebia rufa*	229	EY, VIII.2022	BLS M-3565/EYu 220802-12	BPF, *Corylus avellana*
*Ph. rufa*	232	EY, V.2022	BLS M-11610/EYu 230501-52	BPF, *Fraxinus excelsior*
*Phlebiodontia* cf. *subochracea*	313	EY, MW, IX.2023	BLS M-10846/EYu 230919-7	Łuków vicinities, *Abies alba*
*Phlebiopsis gigantea*	247-1	EY, KW, XII.2022	BLS M-3977/EYu 221229-4	Hajnówka vicinities, *Pinus sylvestris*
*Ph. gigantea*	247-2	EY, V.2023	BLS M-10059/EYu 230501-8	BPF, *Pinus sylvestris*
*Scopuloides hydnoides*	338	EY, IX.2023	BLS M-10835/EYu 230926-5	BPF, *Fraxinus excelsior*
*Steccherinum bourdotii*	339	EY, MW, IX.2023	BLS M-10850/EYu 230919-1	Łuków vicinities, *Acer pseudoplatanus*
*Steccherinum ochraceum*	296	EY, VII.2023	BLS M-10315/EYu 230702-2a	BPF, *Carpinus betulus*
*S. ochraceum*	340	EY, IX.2023	BLS M-10837/EYu 230926-3	BPF, *Alnus glutinosa*
Russulales				
*Asterostroma medium*	312	EY, IX.2023	BLS M-10845/EYu 230919-8	Łuków vicinities, *Abies alba*
*Baltazaria galactina*	231	EY, VIII.2022	BLS M-3563/EYu 220802-4	BPF, *Populus tremula*
*B. galactina*	233	MW, IX.2022	BLS M-3981	BPF, *Tilia cordata*
*Dentipellis fragilis*	342	MW, X.2023	BLS M-10828	BPF, *Alnus glutinosa*
*Gloiothele lactescens*	314	EY, IX.2023	BLS M-10841/EYu230921-5	BPF, cf. *Fraxinus excelsior*
*Laxitextum bicolor*	178	MW, IX.2020	BLS M-3630	BPF, *Salix caprea*
*L. bicolor*	235	MW, X.2022	BLS M-3996	Białystok vicinities, *Salix caprea*
*Peniophora cinerea*	293	EY, VI.2023	BLS M-10325/EYu 230625-2	BPF, *Alnus glutinosa*
*P. cinerea*	295	EY, VII.2023	BLS M-10316/EYu 230710-3	BPF, *Corylus avellana*
*P. cinerea*	310	EY, IX.2023	BLS M-10857/EYu 230907-4	BPF, *Sorbus aucuparia*
*Peniophora incarnata*	272	EY, V.2023	BLS M-10062/EYu 230501-5	BPF, *Populus tremula*
*Peniophora laeta*	288-1	EY, V.2023	BLS M-10052/EYu 230509-7	BPF, *Carpinus betulus*
*P. laeta*	288-2	EY, VIII.2023	BLS M-10324/EYu 230803-2	BPF, *Carpinus betulus*
*Peniophora limitata*	290	EY, V.2023	BLS M-10327/EYu 230509-8	BPF, *Fraxinus excelsior*
*Peniophora pithya*	287	EY, V.2023	BLS M-10047/EYu 230509-2	BPF, *Picea abies*
*Peniophora quercina*	237	EY, X.2022	BLS M-3957/EYu 221016-9	BPF, *Quercus robur*
*Peniophora rufomarginata*	249-1	MW, XII.2022	BLS M-10070	BPF, *Tilia cordata*
*P. rufomarginata*	249-2	EY, X.2022	BLS M-3938/EYu 221009-10	BPF, *Tilia cordata*
*Scytinostroma odoratum*	337	EY, MW, IX.2023	BLS M-10849/EYu 230919-3	Łuków vicinities, *Pinus sylvestris*
*Stereum hirsutum*	167	MW, XII.2020		BPF, *Carpinus betulus*
*S. hirsutum*	289	EY, VI.2023	BLS M-10317/EYu 230625-2	BPF, *Alnus glutinosa*
*S. hirsutum*	294-1	EY, VII.2023	BLS M-10308/EYu 230710-1	BPF, *Quercus robur*
*S. hirsutum*	294-2	EY, VIII.2023	BLS M-10320/EYu 230803-3	BPF, *Quercus robur*
*Stereum rugosum*	79	EY, IV.2023	BLS M-10061/EYu 230423-4	BPF, *Corylus avellana*
*S. rugosum*	281	EY, V.2023	BLS M-10065/EYu 230501-3	BPF, *Quercus robur*
*Stereum sanguinolentum*	274	EY, V.2023	BLS M-10055/EYu 230509-1	BPF, *Picea abies*
*S. sanguinolentum*	275	EY, V.2023	BLS M-10058/EYu 230511-6	BPF, *Pinus sylvestris*
*Stereum subtomentosum*	197	MW, X.2019	BLS M-1946	BPF, *Alnus glutinosa*
*Xylobolus frustulatus*	186-1	GK, VI.2021		BPF, *Quercus robur*
*X. frustulatus*	186-2	EY, IV.2023	BLS M-10069/EYu 230423-3	BPF, *Quercus robur*

* EY—Eugene Yurchenko, GK—Grzegorz Kuryło, KW—Konrad Wilamowski, MW—Marek Wołkowycki. ** BPF—Białowieża Primeval Forest, the area between Hajnówka and Białowieża, including Ladzka Primeval Forest.
